# Analysis of Animal Models of Traumatic Osteonecrosis of the Femoral Head Based on Blood Supply: A Literature Review

**DOI:** 10.1111/os.14352

**Published:** 2025-01-21

**Authors:** Linbao Wang, Yunzhuan Luo, Xing Qiu, Liangliang Cheng, Kaiming Ma, Jianchen Guan, Yuchen Liu, Jiawei Ying, Dewei Zhao

**Affiliations:** ^1^ Department of Orthopaedic Affiliated ZhongShan Hospital of Dalian University Dalian China; ^2^ Department of Orthopaedic Affiliated Traditional Chinese Medicine Hospital of Southwest Medical University Luzhou China

**Keywords:** animal model, femoral neck fracture, inflammatory response, retinacular vessels, traumatic osteonecrosis of the femoral head

## Abstract

Traumatic osteonecrosis of the femoral head (TONFH) refers to ischemic osteonecrosis is resulting from an acute mechanical interruption of the blood supply to the femoral head. The early diagnosis and optimal treatment have been central focuses of research and continue to undergo improvement. Reliable animal models are essential for advancing research into the treatment of the disease. This review aims to provide a comprehensive overview of tetrapod models (rats, rabbits, dogs, and sheep) and bipod models (emus, ostriches), as well as various modeling methods (traumatic hip dislocation, dissection of the round ligament and ligature of the femoral neck, femoral neck fracture (FNF), reduction and internal fixation after femoral neck fracture, and highly selective disruption of the anterior‐superior retinacular vessels). This review examines the advantages, disadvantages, and applicability of each model. Based on blood flow analysis, it proposes a more reliable direction for TONFH modeling: simulating partial blood flow injury in the context of FNF.

## Graphical abstract

We mainly summarize the research progress of animal models of TONFH based on the blood supply in recent years, including the advantages and disadvantages and applicability, and speculated a more reliable TONFH modeling direction: to simulate the partial of blood flow injury based on femoral neck fracture.

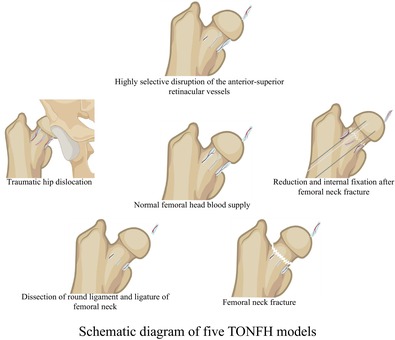

## 1 Introduction

Traumatic osteonecrosis of the femoral head (TONFH) typically results from an acute mechanical interruption of the blood supply to the femoral head, commonly associated with hip trauma such as acute slipped capital femoral epiphysis, intra‐articular femoral neck fractures (FNFs) or posterior hip dislocations [1, 2, 3].

The necrotic location typically occurs in the weight‐bearing area of the femoral head. The weakening mechanical strength of the osteonecrotic trabeculae bone makes it prone to microfracture, which often fail to heal due to the disrupted bone remodeling associated with osteonecrosis. Consequently, these microfractures accumulate over time, ultimately leading to the collapse of the femoral head. Eventually, total hip arthroplasty (THA) becomes the final treatment option [4].

As the world's population aging, the number of global hip fractures is projected to reach approximately 6.3 million by 2050 [5, 6]. FNFs account for approximately 54% of all hip fracture cases [7]. The incidence of FNFs among young and middle‐aged individuals is also increasing [8]. According to relevant literature, the incidence of osteonecrosis after FNFs in young patients can reach as high as 86% [9]. However, TONFH remains an unsolved issue, warranting further investigation.

This review focuses on current research into the blood supply in animal models of TONFH, evaluates their strengths and limitation, and assesses their applicability, thereby offering a foundational framework for future TONFH‐related studies.

## 2 Materials and Methods

### 2.1 Literature Search

**FIGURE 1:**
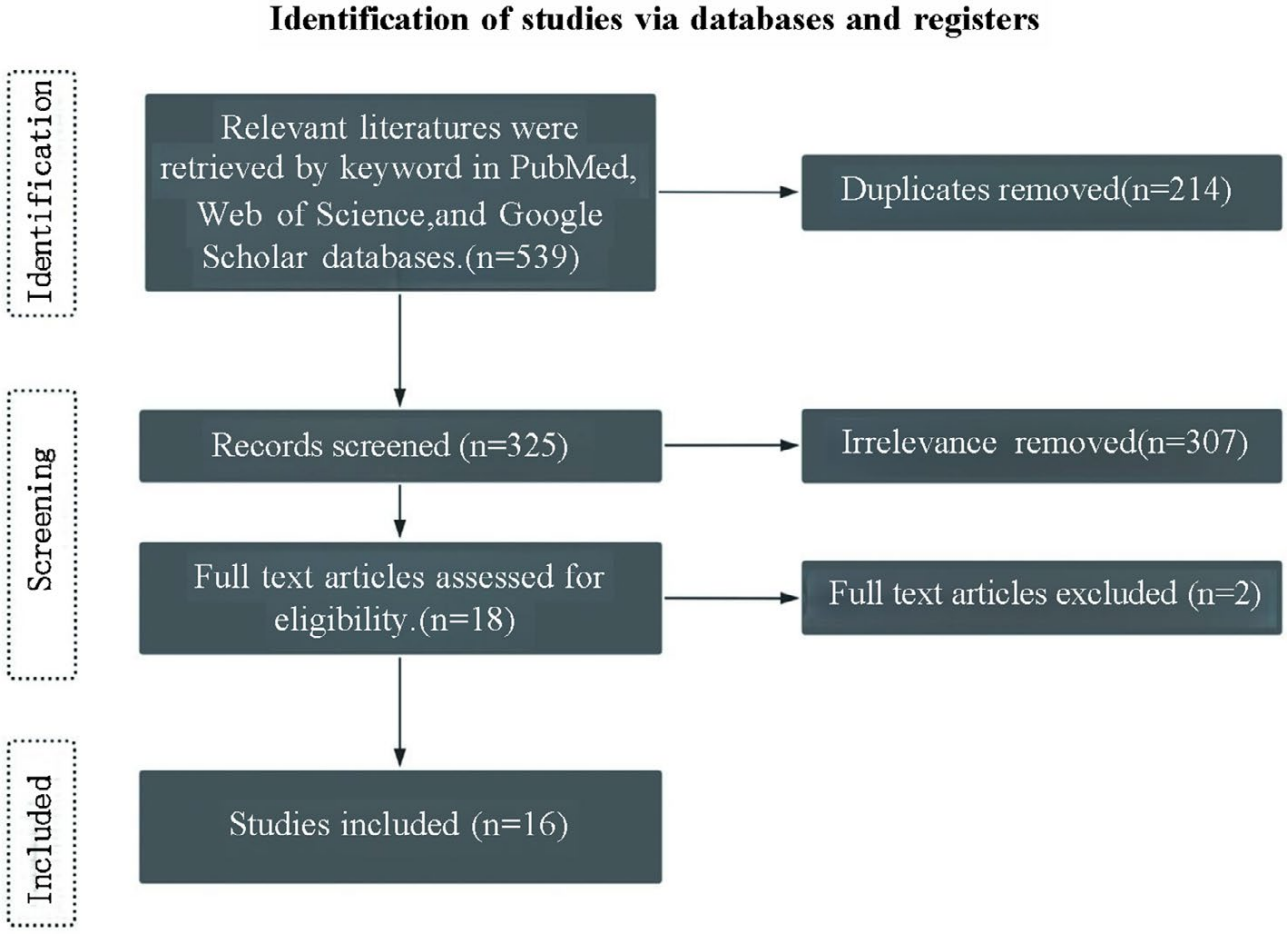
Literature screening flowchart.

The details of the literature search are presented in Figure 1. Relevant articles were retrieved from the PubMed, Web of Science, and Google Scholar databases using the following search terms: (Traumatic osteonecrosis of the femoral head) AND (Animal model), (Femoral neck fracture) AND (Animal model), (Traumatic hip dislocation) AND (Animal model). Similar articles and their associated citations listed under each study were reviewed to identify additional eligible studies, ensuring a comprehensive search. The search was conducted between May 20 and May 30, 2024. Literature published between January 2010 and May 2024 was reviewed, and relevant studies were selected based on predefined inclusion and exclusion criteria. The amount of retrieved literature was 539 articles.

### 2.2 Literature Selection

Inclusion criteria: (i) Published journal papers and reviews. (ii) Studies on animal models of TONFH. (iii) Studies involving blood supply of the femoral head. (iv) Research on the inflammatory response in osteonecrosis of the femoral head (ONFH). Exclusion criteria: (i) Studies not closely aligned with the objectives of this research. (ii) Publications where the full text was unavailable. (iii) Duplicate publications.

### 2.3 Quality Assessment

A total of 539 articles were initially identified. The collectors conducted a preliminary screening by reviewing the titles and abstracts to assess the relevance and validity of the articles for inclusion. Duplicate studies and unrelated articles were excluded, resulting in the final inclusion of 16 articles sourced from the PubMed, Web of Science, and Google Scholar databases (Figure 1).

## 3 Results

### 3.1 Blood Supply to the Femoral Head in Normal and FNFs

The blood supply to the femoral head primarily originates from the superior retinacular artery (SRA), anterior retinacular artery (ARA), inferior retinacular artery (IRA), and the artery of the round ligament (RLA) [10, 11, 12]. The branches of the three retinacular arterial groups form an epiphyseal arterial network above the epiphyseal scar and a metaphyseal arterial network below it. The branches of the RLA integrate with the epiphyseal arterial network, which is the most widely distributed and serves as the primary vascular network structure in the femoral head [10, 13].

The conventional perspective suggests that the extramedullary blood supply is substantially compromised and, in severe cases, might be completely disrupted following FNFs. Consequently, many TONFH models involving vascular disruption have been developed by dissecting the round ligament and ligating the femoral neck. Zhao et al. [10] demonstrated via digital subtraction angiography (DSA) that ARA and SRA are closely attached to the bone surface, rendering them highly susceptible to damage following FNFs. The disruption of the extramedullary blood supply is significantly more severe in displaced FNFs (Garden types III and IV) compared to non‐displaced fractures (Garden types I and II). The epiphyseal arterial network and the IRA are likely the two critical structures responsible for maintaining the blood supply to the femoral head following FNFs. This perspective provides a theoretical foundation for developing a novel animal model to study femoral head blood flow after FNFs.

### 3.2 Histopathology of ONFH

ONFH can be categorized into three stages of blood supply alteration: the early stage (venous stasis period), the middle stage (arterial ischemia period), and the late stage (arterial occlusion period) [14, 15, 16]. In the initial stage of TONFH, the arterial and venous blood flow is disrupted, leading to arterial ischemia and progressively manifesting histological changes characteristic of the middle stage of blood flow alteration [16]. During this stage, venous thrombosis worsens, accompanied by compression‐induced stenosis of arteriovenous vessels or arterial thrombosis, resulting in a significant reduction in the number of arterial vessels [17]. The insufficient blood supply at this stage results in subchondral fractures, expansion of necrotic areas, and localized cystic changes. Partial collapse of the femoral head occurs, and necrotic bone tissue transitions into the repair stage. New blood vessels and fibrous tissue infiltrate the necrotic region, forming granulation tissue. As the disease progresses, it may advance to the late stage of blood supply alteration [15, 16].

### 3.3 Methods of Modeling TONFH

**FIGURE 2:**
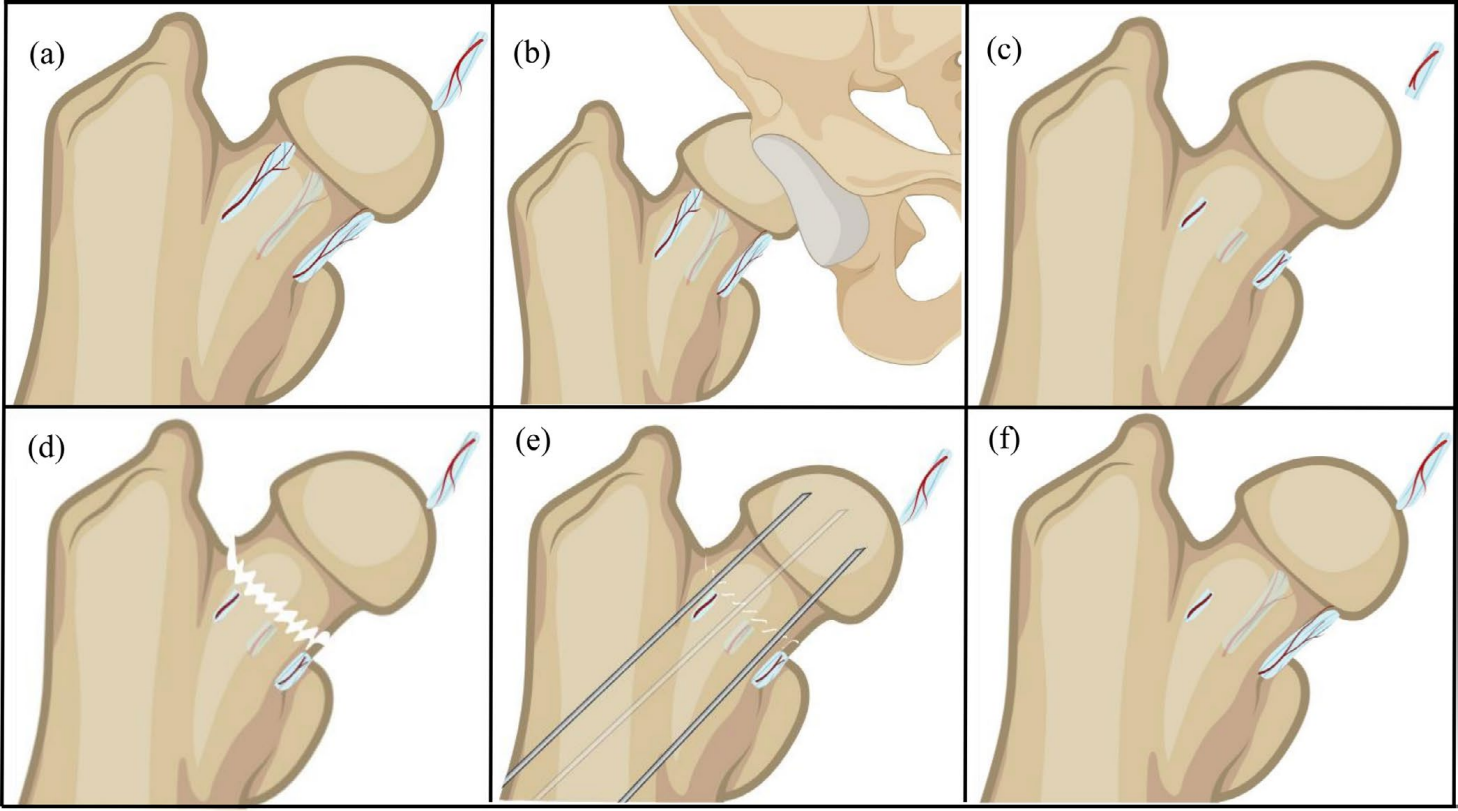
The schematic diagram of TONFH models. (a) Normal femoral head blood supply, (b) traumatic hip dislocation, (c) dissection of the round ligament and ligature of the femoral neck, (d) femoral neck fracture, (e) reduction and internal fixation after femoral neck fracture, and (f) highly selective disruption of the anterior‐superior retinacular blood vessels. This figure was created with BioRender.com.

Animal models of TONFH include tetrapods such as rats, rabbits, dogs, and sheep, as well as bipeds such as emus and ostriches (Table 1). Methods of modeling TONFH include traumatic hip dislocation, dissection of the round ligament and ligature of the femoral neck, femoral neck fracture, reduction and internal fixation after femoral neck fracture, and highly selective disruption of the anterior‐superior retinacular vessels (Table 2). The normal blood supply to the femoral head is illustrated in Figure 2a. The animal models of ONFH induced by alcohol and liquid nitrogen do not replicate the acute mechanical interruption of the blood supply characteristic of clinical cases, nor do they align with the typical pathogenesis observed in patients. Nonetheless, these models remain valuable for studying therapeutic interventions and prognostic factors. Consequently, this review excludes these modeling methods.

**TABLE 1:** Comparison of six TONFH animals.

	Animal	Anatomy of the hip joint	Biomechanics of the hip joint	Blood supply of the femoral head	Other characteristics	Appropriate research	References
Tetrapod	Rat	Similar to humans	Different from humans	Similar to humans	The stress load of both lower limbs can be increased by drinking water at a high level to simulate the biomechanics of the human hip joint.	It is beneficial to the subsequent treatment methods and the efficacy research of early ONFH.	[26, 32]
	Rabbit	Different from humans	Different from humans	Different from humans	—	It is beneficial to the subsequent treatment methods and the efficacy research of early ONFH.	[29, 30], [39, 40, 41]
	Dog	Similar to humans	Different from humans	Similar to humans	The ONFH model with tripod loading can be established to simulate the biomechanics of the human hip joint.	It is beneficial to the subsequent treatment methods and the efficacy research of early ONFH.	[31, 35, 36]
	Sheep	Similar to humans	Different from humans	Similar to humans	—	It is beneficial to the subsequent treatment methods and the efficacy research of early ONFH.	[33, 34, 37, 38]
Biped	Emu and ostrich	Similar to humans	Similar to humans	Similar to humans	The pathologic development and collapse process of ONFH were similar to that of humans.	It is beneficial to study the subsequent treatment methods and the efficacy methods of early, middle and late ONFH.	[42, 43, 44]

**TABLE 2:** Comparison of five types of TONFH models.

Modeling methods	Traumatic size	Blood flow of the femoral head	The round ligament	Advantages	Shortcomings	References
Traumatic hip dislocation	Minor	Partial interruption of the extramedullary blood supply	Dissected. Prone to dislocation.	Preserve some blood supply and restore blood flow after the reduction.	The dislocation and reduction conditions in different individuals may lead to variations in the degree of ischemia, affecting the assessment of the necrosis process.	[24, 25]
Dissection of the round ligament and ligature of the femoral neck	Severe	Complete interruption of the extramedullary blood supply	Dissected. Prone to dislocation.	The success rate of late ONFH modeling is high.	The progression of ONFH is rapid, and it is not conducive to the study and observation of its progression.	[26, 27, 28], [45]
Femoral neck fracture	Severe	Partial/complete interruption of intramedullary and extramedullary blood supply	Dissected. Prone to dislocation.	The success rate of late ONFH modeling is high.	The progression of ONFH is rapid, and it is not conducive to the study and observation of its progression.	[29, 30]
Reduction and internal fixation after femoral neck fracture	Severe	Partial/complete interruption of intramedullary and extramedullary blood supply	Dissected. Prone to dislocation.	The success rate of late ONFH modeling is high.	The progression of ONFH is rapid, and it is not conducive to the study and observation of its progression.	[31]
Highly selective disruption of the anterior‐superior retinacular vessels	Minor	Partial interruption of the extramedullary blood supply	Not dissected. Not prone to dislocation.	A good method to simulate the extramedullary blood flow of the femoral head after FNFS in patients.	The FNF model was not established, and the intramedullary blood supply was not interrupted.	[32]

### 3.4 Traumatic Hip Dislocation

Traumatic hip dislocation can lead to cartilage damage and reduced blood flow to the subchondral bone as a result of acute mechanical forces exerted during the dislocation [18]. The development of avascular necrosis (AVN) of the femoral head is likely linked to vascular damage sustained during the dislocation [19]. Clinical studies emphasize that early reduction of hip dislocation should ideally be performed within 12–24 h to minimize complications [20]. Hougaard and Thomsen [21] and Jasulka et al. [22] suggest that hip reduction should be completed within the first 6 h to reduce the risk of complications. However, Dreinhofer et al. [23] observed that despite hip reduction being performed within the first 6 h, the long‐term risk of developing AVN of the femoral head persists.

The specific surgical procedure for modeling involves a posterior approach to the hip joint, ensuring preservation of the sciatic nerve during muscle dissection. Following the incision of the joint capsule and separation of the ligamentum teres, the hip joint is gently dislocated posteriorly, applying minimal force to avoid spontaneous reduction (Figure 2b). The force applied during dislocation is carefully minimized to mitigate the effects of mechanical trauma on the cartilage. Since there are no obvious fractures associated with the hip dislocation, the risk of vascular damage to the inferior supporting structures is considered minimal. Although mechanical pressure increases and blood supply to the femoral head is reduced, complete interruption of the blood supply does not occur. According to the stage of pathological blood flow changes in ONFH, this corresponds to the middle stage of blood flow changes (arterial ischemia stage).

Using the Elliott et al. [24] model, TUNEL staining identified apoptotic cells predominantly in the superficial and outer regions of the articular cartilage. HE staining demonstrated no significant differences in the chondrocyte morphology or cartilage thickness. Studies from Johanna E et al. [24] and Savaş et al. [25] indicate that prolonged dislocation of the rat hip joint (beyond 1 h) can induce chondrocyte apoptosis. The apoptotic index of chondrocytes after joint dislocation increases in a time‐dependent manner. Early reduction of hip dislocation may therefore mitigate the risk of AVN.

### 3.5 Dissection of the Round Ligament and Ligature of the Femoral Neck

The key to the specific modeling approach involves femoral head dislocation by dissecting the round ligament, followed by electrocoagulation cauterization of the soft tissue surrounding the femoral neck. Alternatively, sutures can be used to ligate the surrounding vessels, effectively interrupting the extramedullary blood supply to the femoral head (Figure 2c). Based on the stage of pathological blood flow changes in ONFH, this TONFH modeling method corresponds to the late stage of blood flow changes (arterial occlusion stage).

Cheng et al. [26] successfully established a rat model of TONFH using this method. The findings demonstrated that the expressions of inflammatory cytokines, including IL1‐β, IL33, and IL17A, were significantly upregulated. Histopathological analysis revealed significant reductions in the height‐to‐diameter ratio of the epiphysis (H/D) and the bone volume‐to‐total volume ratio (BV/TV). Deng et al. [27] utilized a piglet model to induce TONFH. Four weeks post‐modeling, deformation of the femoral head was evident. Pathological examination revealed an increased number of empty bone lacunae, while the marrow space showed infiltration of small blood vessels, fibroblasts, and adipocytes around the necrotic femoral head, accompanied by heightened osteoclast activity involved in the absorption of necrotic bone tissue. Park and Him [28] developed a piglet model of TONFH. Four weeks post‐modeling, a significant increase in trabecular bone mineralization was observed in the subchondral area of the femoral head, with some specimens exhibiting crescent signs and subchondral fractures. No new bone formation on the ischemic side, while bone formation continued on the normal side, exacerbating discrepancies in trabecular structure between the two regions. This disparity in trabecular structure and mechanical load may contribute to the development of subchondral fractures.

### 3.6 Femoral Neck Fracture

FNFs can cause varying degree of damage to the surrounding blood vessels supplying the femoral head. The specific modeling approach involves creating an FNF model by dissecting round ligament and ligating femoral neck (Figure 2d). Based on the stage of pathological blood flow changes in ONFH, this TONFH modeling method corresponds to the late stage of blood flow changes (arterial occlusion stage). In certain studies, the round ligament remained intact, and the femoral neck was not ligated during modeling process. Instead, the model was created directly by inducing an FNF. Consequently, the IRA may remain intact, partially maintaining the blood supply to the femoral head. Based on the stage of pathological blood flow changes in ONFH, this corresponds to the middle stage of blood flow changes (arterial ischemia stage).

Zhang et al. [29] utilized rabbits for their modeling study. After 2 weeks of modeling, the femur on the experimental side showed a white appearance, typical of avascular necrosis. MRI results revealed the presence of a ‘crescent sign’. Additionally, histomorphological analysis further demonstrated that the compromised trabecular bone, an increased number of empty bone lacunae and the signs of osteonecrosis. Wen Q et al. [30] established an FNF model in rabbits without dissecting the round ligament or ligate or electrocoagulating the extramedullary blood vessels of the femoral neck. Postoperative angiography was not performed, making it unclear whether the retinacular vessels were preserved in the model. Three days after modeling, the number of von Willebrand factor (vWF)‐stained microvessels (MVs) decreased rapidly. Two weeks post‐modeling, the number of empty bone lacunae increased and the trabecular thickness decreased. At 3 weeks, CD105, a specific marker of new blood vessels, indicated the presence of a small number of MVs, suggesting ongoing revascularization and tissue self‐repair in the femoral head.

### 3.7 Reduction and Internal Fixation After Femoral Neck Fracture

Effective modeling requires reduction and internal fixation using the FNF model, achieved with three parallel Kirschner pins arranged in an inverted triangle pattern (Figure 2e). Zhao et al. [10] demonstrated that peripheral internal fixation poses a high risk of damaging the main stems of the epiphyseal arteries. The internal fixation method of three parallel cannulated compression screws (CCS) in an inverted triangle pattern can easily damage the epiphyseal arterial network, significantly increasing the risk of AVN of the femoral head. The pathological blood flow stage in this ONFH model corresponds to that of the FNF model.

Gao et al. [31] utilized dogs as animal models for their study. The round ligament remained intact and the femoral head was immediately reduced and fixed using three parallel Kirschner pins in the pattern of an inverted triangle during the operation. The FNF model without internal fixation served as the control group. All fracture models were classified as Garden type IV, characterized by near‐total disruption of the femoral head's blood supply. The typical manifestations of TONFH were observed 8 weeks postoperatively. Morphologically, animals in the fracture fixation group preserved the shape of their femoral heads with similar smoothness and luster. In contrast, the femoral heads in the unfixed fracture group lost its normal contour. Histopathological analysis revealed bone marrow cell debris, empty bone lacunae, and increased fat cells, confirming the successful induction of TONFH.

### 3.8 Highly Selective Disruption of the Anterior‐Superior Retinacular Vessels

**FIGURE 3:**
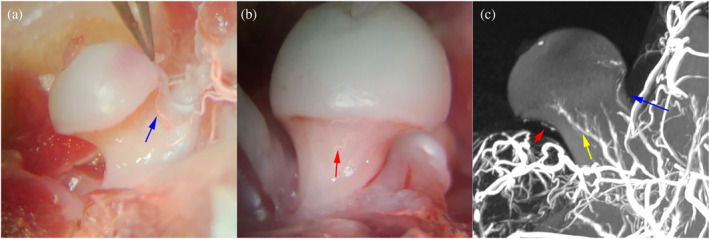
The normal rat femoral head blood supply. The rat femoral head is nourished by the anterior‐superior, inferior and posterior retinacular arteries. The anterior‐superior retinacular branches were the main stems of the femoral head epiphyseal artery. (a) The front view of the left hip showing the anterior‐superior retinacular branches; (b) the inside view of the left hip showing the inferior retinacular branches; and (c) the femoral head blood vessels perfusion and reconstruction showing the anterior‐superior (Blue arrow), inferior (Red arrow), and posterior (Yellow arrow) retinacular arteries [32].

Lv et al. [32] employed a radiopaque barium sulphate suspension to conduct microangiography on the femoral head of rats, followed by micro‐CT scanning. The study identified the main vessels supplying the epiphyseal region of the femoral head: the anterior‐superior retinacular arteries (ASRA), posterior retinacular arteries (PRA), and inferior retinacular arteries (IRA) (Figure 3). Among these, the ASRA form the principal stems of the epiphyseal artery upon entering the femoral head. The ASRA exhibit a consistent anatomical position and a larger diameter compared to the PRA and IRA.

**FIGURE 4:**
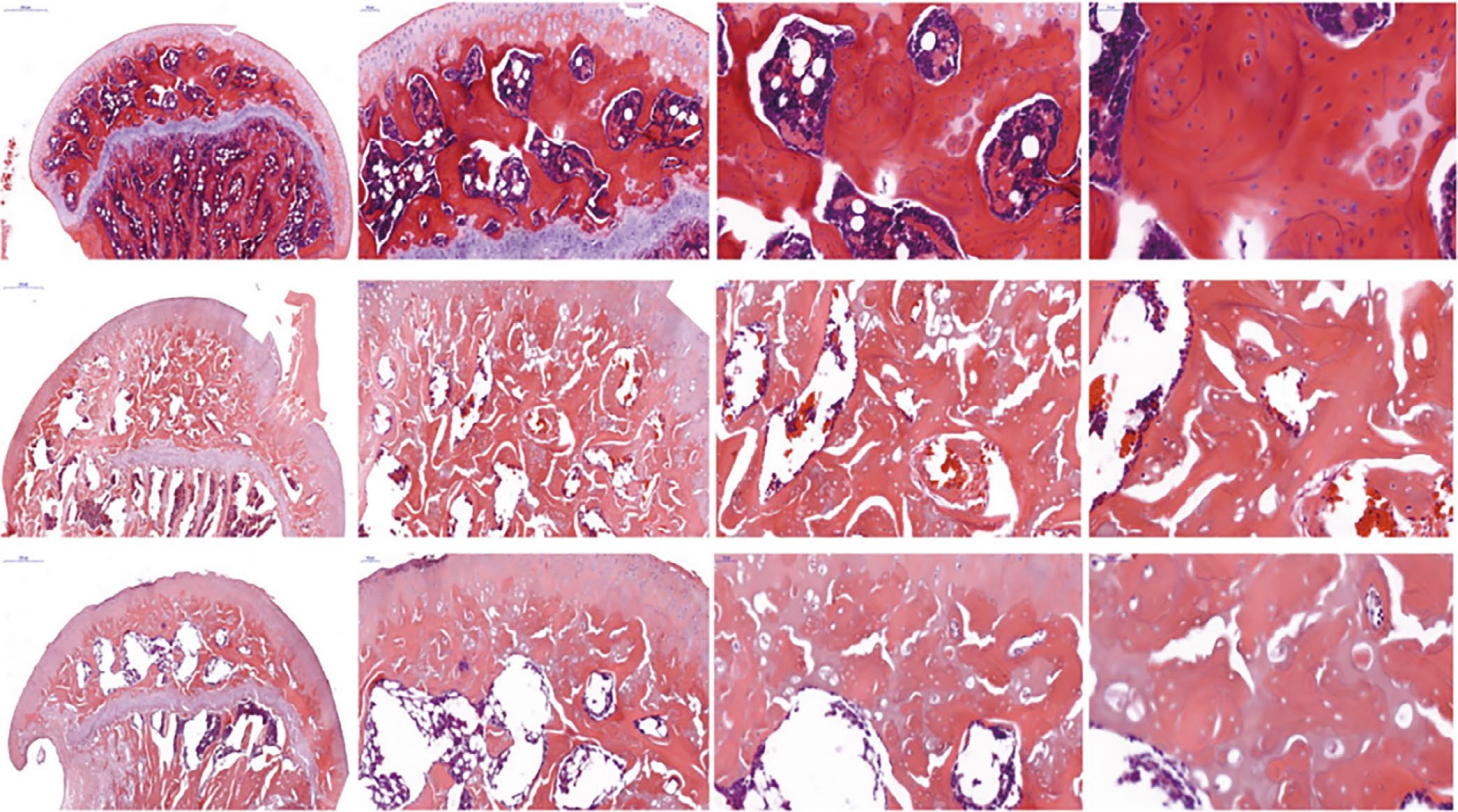
Hematoxylin and eosin staining of rat femoral heads. The deprivation of the anterior‐superior retinacular blood vessels resulted in cell loss in most regions of the femoral head in terms of nuclear staining in the trabeculae and bone marrow; the structure was disorganized in the femoral head epiphysis after 5 and 10 weeks; however, the entire femoral head epiphysis did not appear completely necrotic. Chondral surfaces were not round and smooth in the ischemic group at week 10. In contrast, the chondral surface was round and smooth, and no cell death or bone structure disorganization manifested in the control group [32].

This investigator employed microsurgical instruments under the microscope to identify and highly selectively block the ASRA supplying the femoral head of rats using bipolar electrocoagulation, thereby establishing a partial vascular deprivation‐induced TONFH rat model (Figure 2f) [32]. During the modeling process, the remaining retinacular arteries and the round ligament were preserved. Based on the stage of pathological blood flow changes in ONFH, this corresponds to the middle stage of blood flow changes (arterial ischemia stage). Histopathological analysis revealed the loss of osteocyte nuclear staining in the majority most bone marrow cells (Figure 4). After 5 weeks, histopathology showed disordered trabecular structures, a significant reduction in BV/TV, deformation of the femoral head epiphysis, and a decreased H/D ratio. By the 10th week, trabecular thickness had significantly decreased, and the H/D ratio further declined. The results demonstrate that highly selective blocking of the ASRA effectively induced TONFH in rats.

## 4 Discussion

### 4.1 The Selection of Animal Models

In hip trauma, TONFH is one of the major complications of FNFs [5]. Its etiology is complex, and its pathogenesis has not been fully defined, making it one of the research hotspots at home and abroad. A reliable animal model is needed to study the disease. The early diagnosis and optimal treatment are the prominent focuses of research both domestically and internationally. Developing a reliable animal model is essential for advancing the study of this disease.

Animal models currently used to study TONFH include tetrapods (rats, rabbits, dogs, and sheep) and bipeds (emus and ostriches) (Table 1). Although the anatomical structure and blood supply of the hip joint in rats, dogs, and sheep are similar to those in humans, their biomechanical characteristics differ significantly, making it challenging to develop a reliable model for femoral head collapse. Therefore, this animal model is particularly suited for studying early‐stage ONFH, which facilitates subsequent research on treatment strategies and efficacy [30, 32, 33, 34, 35, 36, 37, 38]. However, the anatomy, biomechanics, and blood supply of the hip joint in rabbits differ significantly from those of humans [39, 40, 41]. Bipedal animals such as emus and ostriches share similarities with humans in height, weight, and hip joint anatomy, as well as biomechanical characteristics and the pathological progression of ONFH. Therefore, using emus and ostriches as models offers distinct advantages for studying ONFH [42, 43, 44].

### 4.2 The Current Challenges of Establishing the TONFH Model

Traditionally, it is believed that the extramedullary blood flow to the femoral head is severely disrupted following FNFs. Current methods of modeling TONFH include traumatic hip dislocation, dissection of the round ligament and ligature of the femoral neck, femoral neck fracture, and reduction and internal fixation after femoral neck fracture, etc. (Table 2). Traumatic hip dislocation: This involves minor trauma, causing a temporary partial interruption of the extramedullary blood supply, which is typically restored after reduction [24, 25]. The method is simple and straightforward, offering a model that closely resembles acute traumatic dislocations with minimal tissue injury. It is suitable for studying the pathophysiology of traumatic dislocation and the effect on joint stability. Dissection of the round ligament and ligature of the femoral neck: This method involves severe trauma, leading to complete disruption of the extramedullary blood supply [26, 27, 28, 45]. This approach provides a reliable model for simulating femoral head ischemia, as it completely disrupts the extramedullary blood supply. It is particularly useful in studies focused on ischemic necrosis and bone remodeling following blood supply interruption. Femoral neck fracture: This model builds on previous methods by incorporating a fracture of the femoral neck [29, 30]. This method is widely used for studying bone fracture healing and regeneration, especially in models involving elderly populations or those with underlying bone disorders. It allows for clear observation of fracture healing, complications, and surgical interventions. Reduction and internal fixation after femoral neck fracture: This approach involves significant trauma, and the epiphyseal artery network is easily damaged. The intramedullary and extramedullary blood supply is completely disrupted [31]. This method simulates clinical interventions and allows for the study of fracture healing under controlled conditions. It is particularly useful in evaluating the outcomes of internal fixation methods and the prevention of complications like avascular necrosis.

The disease progression of the last three modeling methods is fast, and the success rate of advanced ONFH modeling is high, which is not conducive to the relevant research and observation of the disease progression [26, 27, 28, 29, 30, 31, 45]. The new blood flow view of the femoral head suggests that the three groups of retinacular arteries may be preserved after FNFs supplying blood to the entire femoral head through the epiphyseal artery network [10]. Therefore, Lv et al. [32] proposed a new modeling method: highly selective disruption of the anterior‐superior retinacular vessels, which more accurately simulates the extramedullary blood flow status of the femoral head after FNFs. This method minimizes trauma while partially disrupting the extramedullary blood supply. It is ideal for studies investigating the localized impact of blood supply partial disruption on bone viability and potential therapeutic interventions for ischemia. However, the FNF model was not established, and the intramedullary blood supply was not disrupted.

Each of the aforementioned modeling methods has inherent limitations. Therefore, a new TONFH animal model is needed to reliably simulate the intramedullary and extramedullary partial blood flow injury, reflecting the characteristics of retinacular vessel damage following FNFs [10].

### 4.3 The Outcome of TONFH Ischemia: The Inflammatory Response

Vascular endothelial cells are a highly active endocrine system that can secrete a series of vasoactive substances, regulate vascular tension, and participate in the growth and remodeling of new blood vessels [46]. The relationship between bone and blood vessels is crucial. Osteocytes cannot survive more than 100 mm away from blood vessels [15]. Damage to the bone microvascular endothelial cells directly affects the proliferation and viability of osteocytes. Dysfunction of bone marrow microvascular endothelial cells disrupts the bone marrow microenvironment, potentially contributing to the development of ONFH [47]. Elevated levels of reactive oxygen species (ROS) in the blood can promote the infiltration of inflammatory cells and inhibit the activity of microvascular endothelial cells. The oxidative stress response due to high concentrations of ROS can also directly cause damage to microvascular endothelial cells [48, 49]. Injury to microvascular endothelial cells compromises the vascular wall, causes blood extravasation, increases intraosseous pressure, and results in edema and necrosis of the femoral head [50].

Clinical studies on FNFs revealed that even in non‐displaced FNFs, where retinacular vessels remain intact, the incidence of ONFH is still as high as 25.0% [51, 52]. Following FNFs, the epiphyseal arterial network and the IRA may remain intact. Additionally, the integrity of the hip joint capsule is usually intact. However, fractures can cause rupture of the retinacular vessels, which are the primary blood supply to the epiphysis, along with tissue edema, vascular spasms or kinking, and elevated intracapsular pressure, potentially compressing the IRA and reducing blood flow to the femoral head. Consequently, portions of the bone tissue may enter an ischemic state [1].

Ischemia results in inadequate oxygen supply to bone tissue, leading to damage and death of osteocytes [53]. Damaged osteocytes release various inflammatory mediators, such as cytokines and chemokines. These mediators not only signal cellular damage but also recruit immune cells to the site of injury, initiating a localized inflammatory response. The subsequent infiltration of inflammatory cells further exacerbates local tissue damage, establishing a vicious cycle that amplifies bone cell injury, promotes necrosis, and further impairs blood supply [54].

The activation of the inflammatory response is closely linked to vascular function. Ischemic conditions disrupt the homeostasis of the bone microvascular endothelial cell microenvironment, leading to increased microvascular permeability and hemodynamic alterations [55] These changes promote the infiltration of inflammatory cells into the damaged tissue, thereby exacerbating the localized inflammatory response. Additionally, tissue edema and elevated pressure caused by ischemia can compress surrounding blood vessels, further diminishing blood flow and aggravating the ischemic condition.

### 4.4 Conclusion and Outlook

Although the etiology and pathogenesis of TONFH are well understood, existing animal models exhibit notable limitations. These models fail to reliably replicate the specific histological and morphological alterations observed in human TONFH. The highly selective disruption of the anterior‐superior retinacular vessels offers a promising approach to simulating post‐fracture blood supply changes. However, a fully developed fracture‐based model has not been constructed, and existing models struggle to accurately simulate partial blood supply injuries. The key challenges include the following: (1) Investigating the relationship between ischemia severity (or vascular damage) and the size and location of osteonecrosis. (2) Overcoming the limitations of current techniques (DSA, MRI, DEC‐MRI, etc.) for evaluating femoral head blood supply and predicting TONFH, particularly following FNFs. (3) Designing straightforward and effective treatment strategies for large‐area, early‐stage TONFH. Consequently, addressing these challenges requires the development of innovative animal models that can accurately replicate the partial blood supply injuries characteristic of TONFH, thereby offering a more reliable platform for exploring potential therapeutic interventions.

## Author Contributions

Linbao Wang and Xing Qiu: Conceptualization, methodology, and data curation. Linbao Wang and Yunzhuan Luo: Writing – original draft. Kaiming Ma, Jianchen Guan, and Yuchen Liu: Software and data curation. Liangliang Cheng and Jiawei Ying: Investigation. Liangliang Cheng and Deiwei Zhao: Supervision, project administration, and Writing – review and editing.

## Conflicts of Interest

The authors declare no conflicts of interest.
